# LCA of Cement with Alternative Additives: Pathways to Sustainable Production

**DOI:** 10.3390/ma18133057

**Published:** 2025-06-27

**Authors:** Natalia Generowicz-Caba, Joanna Kulczycka

**Affiliations:** 1 Mineral and Energy Economy Research Institute Polish Academy of Sciences, Wybickiego 7A Str., 31-261 Cracow, Poland; 2 Faculty of Management, AGH University of Krakow, 30-067 Cracow, Poland; kulczycka@min-pan.krakow.pl

**Keywords:** cement, LCA, life cycle assessment, alternative materials

## Abstract

The cement industry is responsible for approximately 7–8% of global CO_2_ emissions, primarily due to the energy-intensive production of clinker. In response to growing environmental concerns and the pressure to decarbonize the construction sector, the composition of cement has been evolving toward more sustainable alternatives. This article presents a review of the recent literature and EPD reports concerning changes in cement composition and their environmental impact, as assessed through Life Cycle Assessment (LCA) methodologies. This paper reviews the literature of recent LCA studies on cement with alternative materials. For a thorough analysis, VOSviewer_1.6.18 was used to find the research gap in this field. The companies’ EPD reports were analyzed to compare the most relevant information. The data that was extracted from the reports concerns carbon footprint, energy consumption, and system boundaries. The analysis reveals a clear trend toward reducing clinker content by incorporating supplementary cementitious materials (SCMs) such as fly ash, ground granulated blast furnace slag, natural pozzolans, and limestone. These modifications significantly lower key LCA indicators, particularly Global Warming Potential (GWP). Despite the growing number of studies on individual SCMs, there is a lack of integrated reviews comparing their environmental performance within a standardized LCA framework. This study addresses this gap by systematically comparing the environmental profiles of various low-clinker cement types and highlighting the critical role of supplementary cementitious materials selection. The findings confirm that changes in cement formulation are not only occurring but are essential for reducing the environmental footprint of construction materials.

## 1. Introduction

Cement is one of the most important construction materials, serving as a key component of concrete, which is widely used in residential, infrastructure, and industrial construction [1]. Its widespread use stems from its excellent mechanical properties, durability, and versatility [2]. However, cement production has a significant environmental impact, placing the cement industry at the center of global discussions on sustainable development and greenhouse gas emissions reduction [3].

The cement manufacturing process is highly energy-intensive and responsible for considerable carbon dioxide (CO_2_) emissions [4]. These emissions primarily originate from two sources: the combustion of fossil fuels in cement kilns and the calcination of limestone, in which calcium carbonate (CaCO_3_) is transformed into calcium oxide (CaO), releasing CO_2_ [5]. It is estimated that cement production accounts for approximately 7–8% of global CO_2_ emissions [6]. In addition, the manufacturing process generates dust, consumes large amounts of water, and contributes to the depletion of natural resources [7].

The environmental impact of cement does not end at the production stage. The life cycle of cement also includes its transportation, use in construction, and end-of-life processes such as building demolition and material recycling [8]. Life Cycle Assessment of cement allows for a comprehensive evaluation of its environmental impact across all life cycle stages, enabling the identification of key areas where impact reduction is possible [9].

Reducing the environmental impact of cement production necessitates interventions across multiple stages of the production process. One of the most important approaches is reducing energy consumption through the optimization of production processes and the use of alternative fuels such as biomass or industrial waste [10]. Innovative technologies, such as carbon capture and storage (CCS), also offer significant potential for reducing CO_2_ emissions [11]. Additionally, using substitutes in cement production allows for the partial replacement of cement clinker—the most energy-intensive component [12]. Among the most commonly used substitutes are fly ash, ground granulated blast furnace slag, and both natural and synthetic pozzolans [13]. These materials not only reduce CO_2_ emissions but can also improve the technical properties of cement, such as durability and resistance to chemical attacks.

A review of the literature shows that Akintayo et al. focused on the environmental impact of a typical cement plant and highlighted the potential improvements in the process [14]. Vargas et al. discussed the potential environmental impact of using treated tailings (TTs) as SCMs at different replacement levels according to specific performance levels in concrete [15]. Caldas et al. evaluated a new bio-concrete from an environmental perspective, incorporating different types and contents of SCMs [16]. Irshidat et al. investigated the feasibility of using waste ceramic byproducts (WCBs) from the aluminum industry as a cement substitute in mortar production [17]. Li et al. explored the use of eco-friendly mortar with high volumes of diatomite and fly ash in cement production [18]. Tosti et al. compared the potential environmental impacts of current waste management practices for biomass ash with its potential reuse as a secondary cementitious material, identifying critical parameters of the modeled systems [19].

The literature review in this article clearly shows that ongoing research continues to focus on improving cement production technologies and reducing environmental impact. Based on the analysis presented in this article, there is a significant gap in comparative LCA studies of cement incorporating alternative materials. This suggests the need for research that not only assesses CO_2_ emissions but also evaluates the full environmental profile of such materials. This article aims to address that research gap and provide an overview of the current state of LCA research for cement, particularly when produced with the addition of alternative materials.

## 2. Materials and Methods

**Figure 1:**
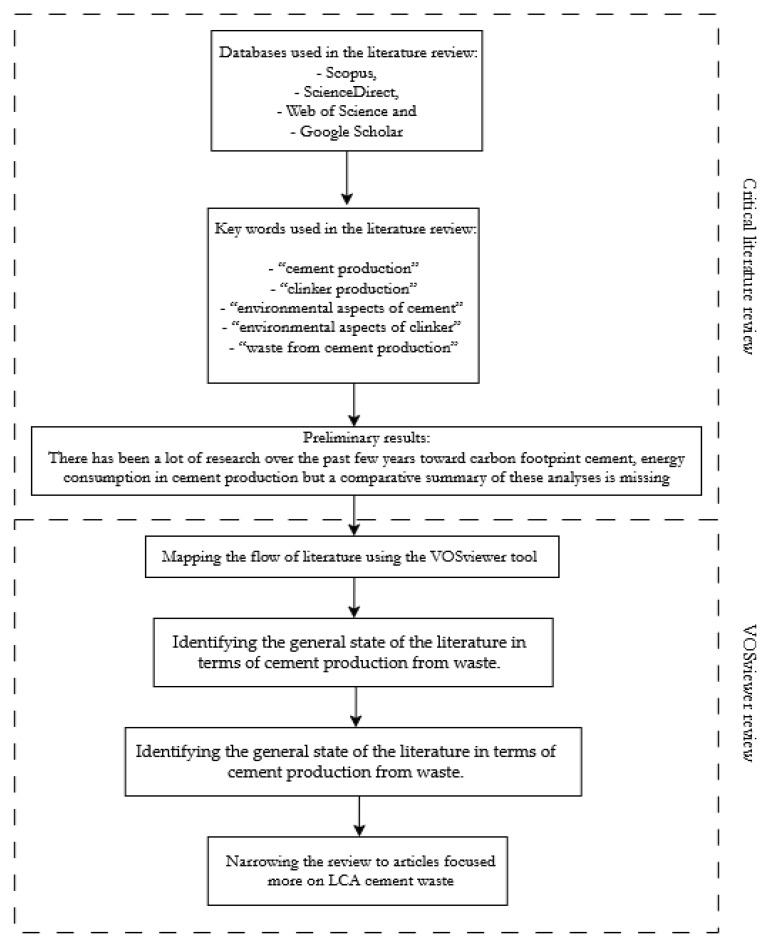
Diagram showing the steps of the literature review.

This article aims to compare life cycle assessment (LCA) studies of cement incorporating alternative materials to determine whether measurable environmental improvements are observed and to identify the factors contributing to such changes. For this purpose, a critical literature review was performed. Figure 1 shows the method undertaken for the literature review.

The review was conducted through a combination of traditional methods for critical literature reviews, internet research, and an analysis of academic multidisciplinary databases such as Scopus, ScienceDirect, Web of Science, and Google Scholar.

The key terms that were used included

- “cement production”, resulting in 13,317 articles across databases;
- “clinker production”, resulting in 1165 articles across databases;
- “environmental aspects of cement”, resulting in 1014 articles across databases;
- “environmental aspects of clinker”, result in 39 articles across databases;
- “waste from cement production”, resulting in 3680 articles across databases.

This led to the preliminary finding that there has been a lot of research over the past few years regarding carbon footprint cement and energy consumption in cement production. Not all of it shows the environmental impact of cement production, but it confirms that this has been a strongly developing topic in recent years as well.

**Figure 2:**
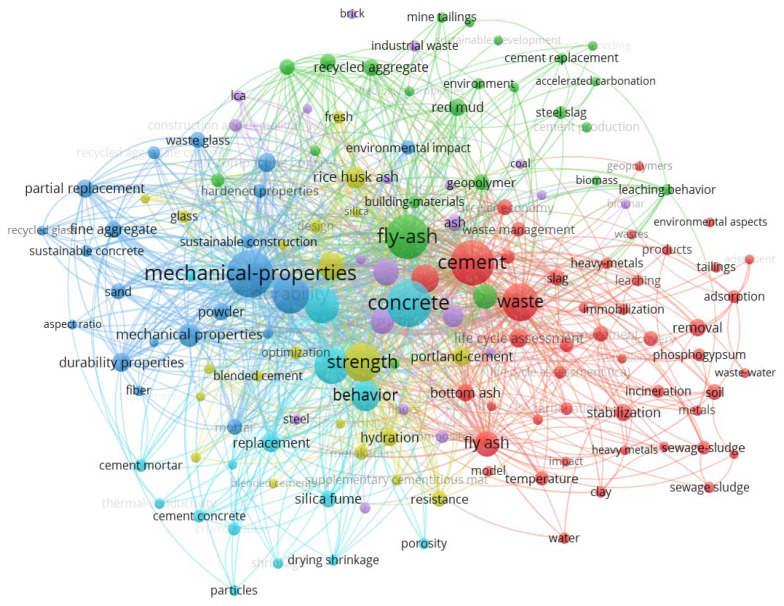
Co-occurrence map of keywords related to “cement from waste environmental aspects”.

The next phase of research involved mapping the flow of the literature. This was performed using the VOSviewer_1.6.18 tool [20], specialized software designed to visualize and construct bibliometric networks that include entities such as journals, researchers, and publications. These networks are based on relationships such as citations, bibliographic links, co-citation, or co-authorship. The mapping process first used the term “cement from waste environmental aspects” (Figure 2) to identify the general state of the literature in terms of cement production from waste. This map identifies dominant research directions and potential research gaps in the literature on the use of waste materials in the cement and concrete industry.

The map uses a clustering method, through which several thematic clusters were identified:

- Blue cluster: This cluster focuses on the mechanical properties and durability of concrete. The main terms are mechanical properties, durability, fine aggregate, replacement, and cement mortar. This cluster reflects the technical aspects of using alternative materials in concrete mixtures.
- Red cluster: This cluster focuses on waste and environmental aspects. Prominent terms include waste, stabilization, adsorption, removal, immobilization, sewage sludge, and heavy metals. This cluster indicates research on the potential use of industrial and municipal waste in cement technology and its impact on the environment.
- Green cluster: This cluster is related to recycling and alternative sources of raw material, including recycled aggregate, sustainable development, red mud, steel slag, and biomass. It includes issues related to the circular economy and the replacement of natural resources with waste.
- Yellow cluster: This cluster deals with material properties and hydration processes. Common words are strength, hydration, behavior, resistance, porosity, and supplementary cementitious materials. This shows interest in the effects of mineral additives on strength and structural properties.
- Turquoise cluster: This cluster contains words related to concrete mix design and aspects of optimization and additives, such as optimization, shrinkage, blended cement, and fly ash.

The size of the individual nodes (keywords) corresponds to their frequency of occurrence, while the thickness of the line represents the strength of co-occurrence between terms. The central position of terms such as concrete, cement, fly ash, waste, strength, and mechanical properties shows their key importance in the analyzed field.

**Figure 3:**
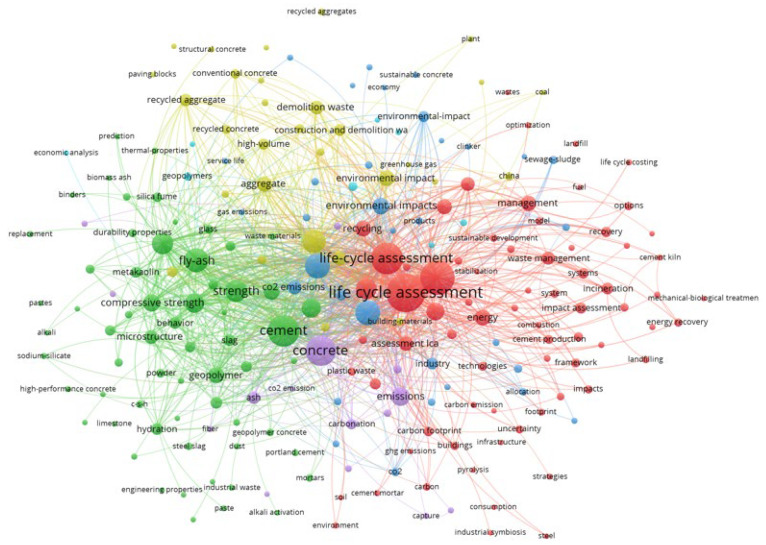
Co-occurrence map of keywords related to LCA cement waste using alternative materials and waste.

The second graph focuses more on publications about “LCA cement waste” (Figure 3). This map identifies the dominant research directions and potential research gaps in the literature on the use of waste materials in the cement and concrete industry.

Keyword analysis identified five main thematic clusters:

- Red cluster (LCA and waste management): The main keywords are life cycle assessment, waste management, management, incineration, energy recovery, and impact assessment. This cluster focuses on the environmental aspects of systemic waste management and their evaluation from an LCA perspective, often in relation to incineration and energy recovery processes.
- Green cluster (alternative materials and mechanical properties): This cluster includes terms such as fly ash, geopolymer, compressive strength, metakaolin, silica fume, slag, and alkali activation. This area is concerned with the use of mineral wastes as additives to cement and concrete and the study of their physical and chemical properties.
- Blue cluster (cement, concrete, and their environmental assessment): This cluster contains central buzzwords such as cement, concrete, environmental impact, recycling, and greenhouse gas. This cluster bridges the gap between materials technology and the environmental assessment of building materials.
- Yellow cluster (recycled aggregate and the circular economy): This cluster includes terms like recycled aggregate, construction and demolition waste, structural concrete, and sustainable concrete. It focuses on aspects of the recovery and reuse of aggregates from building demolition.
- Purple cluster (emissions and carbon footprint): This cluster includes words such as carbon footprint, carbon emissions, GHG emissions, soil, capture, cement production, and carbonation. It focuses on emission analysis and mitigation strategies in the cement industry.

Based on a review of the literature, it is noted that there is a lack of studies that compare different waste additive options (e.g., fly ash vs. metakaolin vs. steel slag) in a single, comparative LCA analysis. This suggests that the literature has largely focused on single-case analyses, with no attempts to cross-reference them in terms of the full product life cycle.

**Figure 4:**
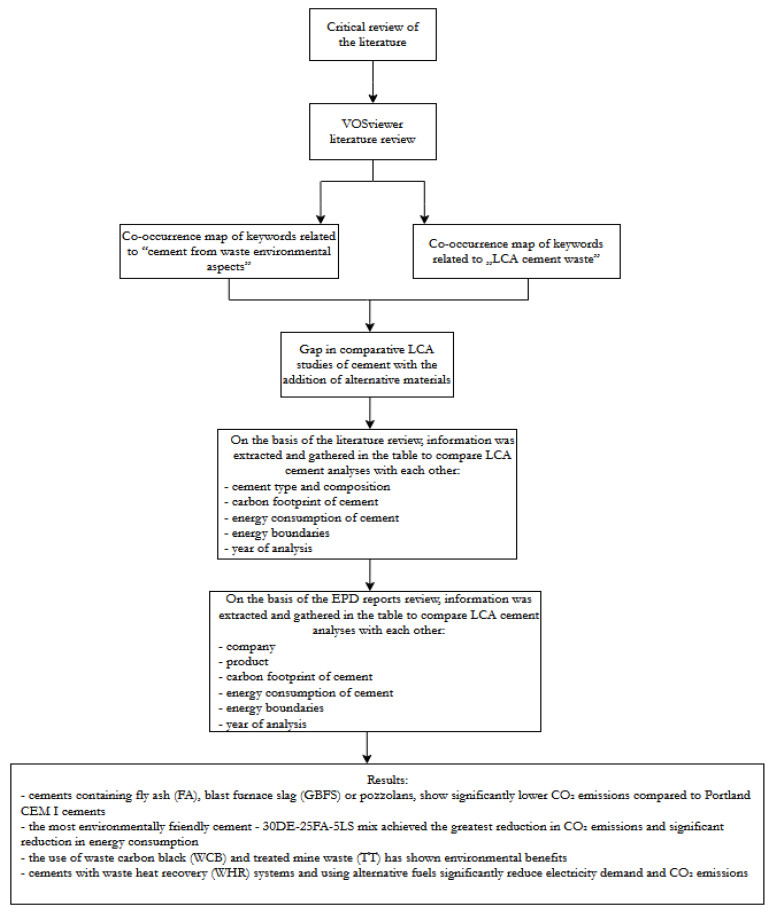
Graphical representation of the research methodology.

The next step was to prepare a compilation of the found publications and reports and compare key information with when analyzing the LCA. The following diagram (Figure 4) shows the algorithm of action when it comes to collecting the data needed to compare LCA studies.

The review as a whole was based on available reports and publications from 2020 to 2025 in order to present the most recent research.

## 3. Results

In the first stage, key data were collected from scientific articles. The data included cement type, carbon footprint, energy consumption, and system boundaries. All of the information was collected in one table (Table 1) with an explanation of cement composition. To provide consistency in the comparative assessment, the studies selected for analysis share several common features. Most of them adopt a functional unit of 1 ton of cement or 1 cubic meter of cement-based material, depending on whether the study focused on cement itself or its application in mortar or concrete. Geographically, the publications cover a range of regions including South Africa [14], Europe (e.g., Turkey, Italy, Portugal, Ireland) [15,16,17,18,19,21], and the Middle East [17]. The analyzed products range from traditional Portland cement (CEM I) to low-clinker variants incorporating supplementary cementitious materials such as fly ash, metakaolin, slag, or treated tailings [22,23]. All studies used the cradle-to-gate system boundaries (A1–A3), and the LCA methodologies are aligned with current standards and norms.

The LCA analysis of different types of cements indicates that the greatest environmental benefits come from cements with reduced clinker content and with the addition of alternative materials. Based on the results of CO_2_ emissions and energy consumption, the best options can be distinguished:

- Cements containing a high proportion of mineral additives—in particular, cements containing fly ash (FA), granulated blast furnace slag (GBFS), or pozzolans, which show significantly lower CO_2_ emissions compared to Portland CEM I cements.
- The most environmentally friendly cement—30DE-25FA-5LS mix achieved the greatest reduction in CO_2_ emissions (60%) and a significant reduction in energy consumption (50%) while maintaining high mechanical strength.
- Cements with industrial waste—the use of waste carbon black (WCB) and treated mine waste (TT) has shown environmental benefits, but their effectiveness depends on the level of cement substitution and additional energy consumption in processing these materials.
- Energy efficiency—cements with waste heat recovery (WHR) systems and using alternative fuels significantly reduce electricity demand and CO_2_ emissions.

In conclusion, the best solution from the point of view of sustainability is to use cements with a high content of substitute materials, while minimizing additional energy consumption and optimizing production processes.

The second stage focused on the available EPD reports, from which we also extracted key data such as the type of cement, the carbon footprint of the cement, energy consumption during its production, the limits of the system, and the year the report was made. Table 2 shows the carbon footprint of companies’ EPD reports with the energy consumption of cement production.

**Figure 5:**
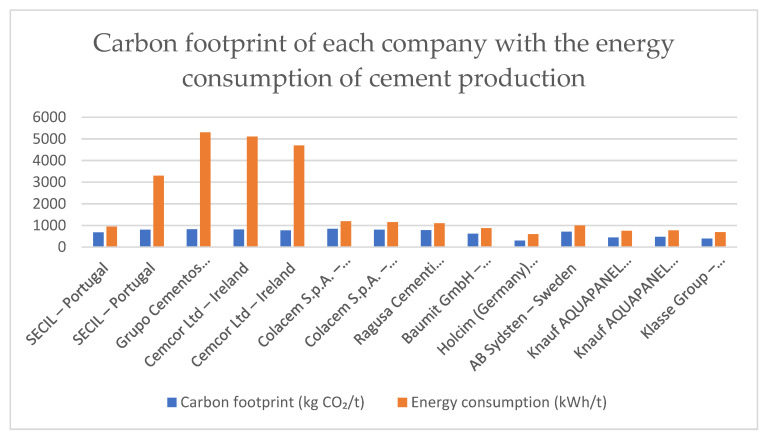
Carbon footprint of companies’ EPD reports with the energy consumption of cement production.

Figure 5 shows the carbon footprint of companies’ EPD reports with the energy consumption of cement production.

Based on an analysis of the chart illustrating the carbon footprint and energy consumption of various cement producers, compiled from Environmental Product Declarations (EPDs), significant differences in the environmental efficiency of individual companies can be identified.

The lowest carbon footprint associated with the production of one ton of cement was recorded for Knauf AQUAPANEL, with carbon dioxide emissions amounting to approximately 530–550 kg CO_2_/t. This value is significantly lower than that of the remaining producers. On the opposite end, CEMCOR Ltd., Ireland, exhibits a carbon footprint exceeding 930 kg CO_2_/t, making it the most emission-intensive producer within the analyzed group. Such a high level of emissions may be attributed to a high clinker content in the cement and limited use of low-emission technologies and fuels.

In terms of energy consumption, Knauf AQUAPANEL also performs most favorably, with the production process requiring approximately 1500–1600 kWh/t. This low energy use indicates high energy efficiency in the technological process. In contrast, Grupo Cementos La Unión shows the highest energy consumption, reaching approximately 5600–5700 kWh/t.

## 4. Discussion

The reviewed studies indicate a clear shift in cement production strategies toward reducing environmental impact through the incorporation of supplementary cementitious materials (SCMs) and reductions in clinker content. This transition is primarily driven by the significant contribution of clinker production to CO_2_ emissions and energy demand. Low-clinker cement formulations, such as CEM II, CEM III, and CEM V, consistently demonstrate lower Global Warming Potential (GWP) and Cumulative Energy Demand (CED) when compared to traditional CEM I cement [14,23].

Among the SCMs evaluated, fly ash (FA) and ground granulated blast furnace slag (GGBS) emerge as particularly effective in mitigating environmental impacts [16,18,22]. These materials can reduce CO_2_ emissions by up to 40–80% [18,22], while also contributing to the enhanced durability and resistance of cement-based composites [16,22]. Natural pozzolans, such as metakaolin and volcanic ash, offer similar benefits, especially in regions where they are readily available [16,18]. Limestone, though less reactive, plays an important role as a filler that contributes to clinker reduction, thereby lowering energy consumption [18]. Additionally, biomass ashes—including those derived from rice husk, wood, or paper sludge—are increasingly recognized as viable low-emission SCMs [18,19].

A key factor influencing the environmental performance of SCM-based cement is regional availability and the associated transport and processing impacts. The reviewed LCA studies suggest that optimizing SCM selection based on local material streams can significantly enhance overall sustainability outcomes [15,16,19]. Therefore, regionalization and material circularity should be incorporated into future cement decarbonization strategies.

Despite the environmental benefits, the implementation of SCMs in cement formulations faces practical barriers. Differences in national technical standards, limited market acceptance, and the lack of harmonized environmental data all contribute to slow adoption [17,21]. Several studies have highlighted the need for standardized LCA methodologies that account for performance-based metrics and regional conditions to ensure the comparability and reliability of results [15,16,18].

Moreover, the regulatory environment and economic incentives play a crucial role in promoting the adoption of low-clinker cements. Policies supporting carbon pricing, public procurement criteria based on environmental performance, and investments in low-carbon infrastructure can significantly accelerate the transition toward more sustainable cement production [23].

In summary, the findings of this review underscore the need for a coordinated approach that combines material innovation, regional SCM strategies, robust LCA frameworks, and supportive policy mechanisms to achieve significant reductions in the environmental footprint of cement.

## 5. Conclusions

The analysis of the reviewed LCA studies reveals several key findings and trends in the development of sustainable cement production.

- Reducing clinker content is a primary strategy for lowering CO_2_ emissions and energy use. Blended cements (CEM II–IV), which incorporate supplementary cementitious materials (SCMs), are replacing traditional CEM I due to their significantly lower environmental impacts [14,16,18,23].
- Fly ash and slag are the most effective SCMs, enabling CO_2_ reductions of up to 60–80%. Other materials like metakaolin, limestone, and biomass ashes (e.g., wood or rice husk ash) also show environmental potential, especially when locally sourced [16,18,19,22].
- The regional availability of SCMs strongly influences environmental performance. Proximity to sources like power plants or steel mills reduces transport emissions and enhances circularity [15,16,19].
- Despite environmental benefits, implementation barriers remain. These include variability in SCM properties, a lack of harmonized LCA methods, and conservative design standards [15,17,18,21].
- Policy support is essential to accelerate the shift. Instruments such as carbon pricing, green procurement, and mandatory EPDs can enable broader adoption of sustainable cement technologies [23].
- Further research is needed on the long-term performance of high-SCM concretes and on comparative, region-specific LCA to guide optimized formulations [16,19,22].

## Figures and Tables

**Table 1:** Comparison of results of LCA analyses for carbon footprint and energy consumption.

Cement Type	Carbon Footprint	Energy Consumption	System Boundaries	Source
Portland Cement produced in South Africa	772 kg CO_2_-eq/t cement	-	Cradle-to-gate A1–A3	[14]
^1^ OPC (without additives, BC-34)	380.3 kg CO_2_/t cement	836 kWh/t TT	Cradle-to-gate A1–A3	[15]
^1^ Cement with TT (OP1-34)	320.97 kg CO_2_/t cement	836 kWh/t cement		
^1^ Cement with TT (LS5-34)	357.66 kg CO_2_/t cement	951 kWh/t cement		
^1^ Cement (BC-41, maximum strength)	455.2 kg CO_2_/t cement	-		
^1^ Cement with TT (OP1-41)	337.72 kg CO_2_/t cement	836 kWh/t cement		
^1^ Cement with TT (LS5-37)	364.57 kg CO_2_/t cement	951 kWh/t cement		
^1^ TTs (treated tailings)—processed copper mining waste used as a substitute material for cement. TT energy consumption includes grinding and calcination processes. System boundaries include the full production process up to the concrete plant exit, excluding use and end of life phases.				
^2^ 100% CEM	415 kg CO_2_-eq/m^3^ WBC	836 kWh/m^3^ WBC	Cradle-to-gate A1–A3	[16]
^2^ 40% MK	320.97 kg CO_2_-eq/m^3^ WBC	951 kWh/m^3^ WBC		
^2^ 30% MK-10% FA	337.72 kg CO_2_-eq/m^3^ WBC	836 kWh/m^3^ WBC		
^2^ 20% MK-20% FA	364.57 kg CO_2_-eq/m^3^ WBC	951 kWh/m^3^ WBC		
^2^ 40% MK-10% FA	Lowest: −39 to 41 (depending on fuel) kg CO_2_-eq/m^3^ WBC	1014 kWh/m^3^ WBC		
^2^ 30% MK-20% FA	100 kg CO_2_-eq/m^3^ WBC	1014 kWh/m^3^ WBC		
^2^ 25% MK-25% FA	−39 (lowest, using wood chips) kg CO_2_-eq/m^3^ WBC	1014 kWh/m^3^ WBC		
^2^ SCM: supplementary cementitious materials (metakaolin—MK; fly ash—FA). Lowest climate impact: the 25 MK-25 FA blend achieved −39 kg CO_2_-eq/m^3^ due to CO_2_ sequestration in wood (scenario using wood chips as fuel for kaolin calcination).				
^3^ 0%(control)	659 kg CO_2_-eq/m^3^	2520 MJ/m^3^	Cradle-to-gate A1–A3	[17]
^3^ 5% WCB	632 kg CO_2_-eq/m^3^	2430 MJ/m^3^		
^3^ 10% WCB	600 kg CO_2_-eq/m^3^	2340 MJ/m^3^		
^3^ 20% WCB	527 kg CO_2_-eq/m^3^	2150 MJ/m^3^		
^3^ 30% WCB	472 kg CO_2_-eq/m^3^	2010 MJ/m^3^		
^3^ 40% WCB	422 kg CO_2_-eq/m^3^	1900 MJ/m^3^		
^3^ WCB: waste carbon black—waste carbon black from the aluminum industry. System boundaries: stages from raw material acquisition (A1) and transportation (A2) to cement mortar production (A3). LCA methodology: the CML 2016 method was used, with analysis of impact categories GWP, AP, EP, and ADP. Best compromise: a mix with 10% WCB, which provided a 9% reduction in CO_2_ emissions while maintaining comparable strength to the control mix.				
^4^ BC-34 (bez TT)	659 kg CO_2_-eq/m^3^	700 kWh/m^3^	Cradle-to-gate A1–A3	[21]
^4^ OP-34 (OP1 TT, 27,8%)	580 kg CO_2_-eq/m^3^	836 kWh/m^3^		
^4^ LS-34 (LS5 TT, 14,4%)	612 kg CO_2_-eq/m^3^	951 kWh/m^3^		
^4^ BC-41 (without TT)	720 kg CO_2_-eq/m^3^	750 kWh/m^3^		
^4^ OP-41 (OP1 TT, 20%)	610 kg CO_2_-eq/m^3^	836 kWh/m^3^		
^4^ BC-20 (without TT)	410 kg CO_2_-eq/m^3^	650 kWh/m^3^		
^4^ OP-20 (OP1 TT, 43,9%)	430 kg CO_2_-eq/m^3^	1014 kWh/m^3^		
^4^ LS-20 (LS5 TT, 40%)	460 kg CO_2_-eq/m^3^	1014 kWh/m^3^		
^4^ TTs (treated tailings): copper mine waste after mechanical and heat treatment. OP1: waste from an operating mine (calcination at 600 °C). LS5: waste from a deposited mine (calcination at 700 °C).				
^5^ 100% GBFS (SCAAC1)	73.6 kg CO_2_-eq/m^3^	1.15 GJ/m^3^	Cradle-to-gate A1–A3	[22]
^5^ 30% FA-70% GBFS (SCAAC2)	60.4 kg CO_2_-eq/m^3^	0.83 GJ/m^3^		
^5^ 40% FA-60% GBFS (SCAAC3)	53.2 kg CO_2_-eq/m^3^	0.72 GJ/m^3^		
^5^ 50% FA-50% GBFS (SCAAC4)	43.9 kg CO_2_-eq/m^3^	0.61 GJ/m^3^		
^5^ 60% FA-40% GBFS (SCAAC5)	33.8 kg CO_2_-eq/m^3^	0.50 GJ/m^3^		
^5^ 70% FA-30% GBFS (SCAAC6)	26.1 kg CO_2_-eq/m^3^	0.39 GJ/m^3^		
^5^ FA (fly ash): fly ash as a substitute for blast furnace slag (GBFS). GBFS (ground granulated blast furnace slag): ground blast furnace slag.				
^6^ 100% PC (control)	2836 kg CO_2_-eq/m^3^	2836 MJ/m^3^	Cradle-to-gate A1–A3	[18]
[18] ^6^ 30FA	2020 kg CO_2_-eq/m^3^	1970 MJ/m^3^		
^6^ 30DE	1900 kg CO_2_-eq/m^3^	1830 MJ/m^3^		
^6^ 30DE-15FA	1700 kg CO_2_-eq/m^3^	1650 MJ/m^3^		
^6^ 30DE-10LS	1600 kg CO_2_-eq/m^3^	1500 MJ/m^3^		
^6^ 30DE-20FA-10LS	1190 kg CO_2_-eq/m^3^	1344 MJ/m^3^		
^6^ 30DE-25FA-5LS	1135 kg CO_2_-eq/m^3^	1418 MJ/m^3^		
^6^ PC: Portland cement; DE: diatomite; FA: fly ash; LS: limestone.				
^7^ OPC (reference)	850 kg CO_2_-eq/t cement	3600 MJ/t cement	Cradle-to-gate A1–A3	[19]
^7^ 20_FA1	705 kg CO_2_-eq/t cement	3150 MJ/t cement		
^7^ 40_FA1	650 kg CO_2_-eq/t cement	3000 MJ/t cement		
^7^ 20_FA2	755 kg CO_2_-eq/t cement	3300 MJ/t cement		
^7^ 40_FA2	722 kg CO_2_-eq/t cement	3180 MJ/t cement		
^7^ 20_FA3	725 kg CO_2_-eq/t cement	3250 MJ/t cement		
^7^ 40_FA3	630 kg CO_2_-eq/t cement	2900 MJ/t cement		
^7^ FA1: ash from a fluidized circulation boiler (clean wood and cocoa shells); FA2: ash from a wood pellet boiler; FA3: ash from a bubbling boiler (paper sludge and waste wood).				
^8^ CEM I (TS)	850 kg CO_2_-eq/t cement	10,985 kWh/t cement	Cradle-to-gate (A1–A3)	[23]
^8^ CEM I (AS)	838 kg CO_2_-eq/t cement	11,467 kWh/t cement		
^8^ CEM II (TS)	766 kg CO_2_-eq/t cement	-		
^8^ CEM II (AS)	721 kg CO_2_-eq/t cement	-		
^8^ CEM IV (TS)	666 kg CO_2_-eq/t cement	-		
^8^ CEM IV (AS)	529 kg CO_2_-eq/t cement	-		
^8^ OWC (TS)	841 kg CO_2_-eq/t cement	-		
^8^ OWC (AS)	864 kg CO_2_-eq/t cement	-		
^8^ Alternative scenario (AS): the use of dried sewage sludge, RDF, and waste oil as alternative fuels, as well as a waste heat recovery (WHR) system.				

Source: own work based on literature review.

**Table 2:** Carbon footprint of companies’ EPD reports with the energy consumption of cement production.

Company Name	Product Name/Type of Cement	Carbon Footprint (kg CO_2_/t)	(kWh/t)	Boundaries of the System	Report Year	Source (Report Name)
AB Sydsten—Sweden (Stockholm)	Betong med Anläggningscement FA	710	1000	Cradle-to-gate (A1–A3)	2020	[24]
Colacem S.p.A.—Italy (Gubbio)	CEM I 52.5 R	850	1200	Cradle-to-gate (A1–A3)	2021	[25]
Colacem S.p.A.—Italy (Rassina)	CEM II/B-LL 32.5 R	810	1150	Cradle-to-gate (A1–A3)	2021	[26]
Ragusa Cementi S.p.A.—Italy (Ragusa)	CEM IV/A(P) 42.5 R–SR	790	1100	Cradle-to-gate (A1–A3)	2021	[27]
Knauf AQUAPANEL GmbH—Germany (Iserlohn)	AQUAPANEL^®^ Cement Board Rooftop 6 mm	450	750	Cradle-to-gate (A1–A3)	2021	[28]
Holcim GmbH—Germany (Hamburg)	Airium™ Spray (cement-based insulation foam)	300	600	Cradle-to-gate (A1–A3)	2022	[29]
Knauf AQUAPANEL GmbH—Greece (Thessaloniki)	AQUAPANEL^®^ Cement Board Outdoor 12.5 mm	480	770	Cradle-to-gate (A1–A3)	2022	[30]
Cemcor Ltd.—Ireland (Cookstown)	CEM I 52.5 N Bulk Cement	819	5100	Cradle-to-gate (A1–A3)	2023	[31]
Cemcor Ltd.—Ireland (Cookstown)	CEM II/A-L 42.5 R Bulk Cement	770	4700	Cradle-to-gate (A1–A3)	2023	[32]
Klasse Group—UK (Swansea)	S-Board (calcium silicate board)	390	690	Cradle-to-gate (A1–A3)	2023	[33]
SECIL—Portugal (Leiria)	CEM II/B-M (V-L) 42.5R (Maceira-Liz)	680	950	Cradle-to-gate (A1–A3)	2024	[34]
SECIL—Portugal (Leiria)	CEM I 52.5R Portland Cement (Outão)	807	3300	Cradle-to-gate (A1–A3)	2024	[35]
Grupo Cementos Portland Valderrivas—Spain (Barcelona)	CEM I 52.5 R Cement (Sevilla)	825	5300	Cradle-to-gate (A1–A3)	2024	[36]
Baumit GmbH—Austria (Vienna)	CEM II/C-M (S-LL) 42.5 N	620	880	Cradle-to-gate (A1–A3)	2024	[37]

Source: own work based on [38].

## Abbreviations

The following abbreviations are used in this manuscript:

AS	Alternative scenario including the use of dried sewage sludge
CCS	Carbon capture and storage
DE	Diatomite
EPD	Environmental Product Declaration
FA	Fly ash
FA1	Ash from a fluidized circulation boiler (clean wood and cocoa shells)
FA2	Ash from a wood pellet boiler
FA3	Ash from a bubbling boiler (paper sludge and waste wood)
GBFS	Ground granulated blast furnace slag
GWP	Global Warming Potential
LCA	Life Cycle Assessment
LS	Limestone
MK	Metakaolin
OPC	Ordinary Portland cement
PC	Portland cement
SCMs	Supplementary cementitious materials
TT	Treated tailing
WCB	Waste ceramic byproducts
WHR	Waste heat recovery system
